# Evidence in Australia for a Case of Airport Dengue

**DOI:** 10.1371/journal.pntd.0001619

**Published:** 2012-09-27

**Authors:** Peter Whelan, Huy Nguyen, Krispin Hajkowicz, Josh Davis, David Smith, Alyssa Pyke, Vicki Krause, Peter Markey

**Affiliations:** 1 Centre for Disease Control, Department of Health, Northern Territory, Australia; 2 Royal Darwin Hospital, Department of Health, Northern Territory, Australia; 3 Menzies School of Health Research, Darwin, Northern Territory, Australia; 4 School of Pathology and Laboratory Medicine, University of Western Australia, Perth, Australia; 5 Public Health Virology, Forensic and Scientific Services, Queensland Health, Queensland, Australia; Duke University-National University of Singapore, Singapore

## Clinical Presentation and Investigation

The Northern Territory of Australia (NT) is currently regarded as free of the vectors of dengue [1]. The vector *Aedes aegypti* was present prior to the 1950s, but disappeared some time between 1956 and 1974, primarily as a result of the widespread reticulation of water during and soon after World War II, and the coincidental removal of rainwater tanks [2]–[4]. Entomologists in the Department of Health operate an exotic mosquito surveillance program to detect any importation or establishment of either *Ae. aegypti* or *Ae. albopictus* [5], and recent incursions of *Ae. aegypti* in two locations within the NT (Tennant Creek in 2004 and on Groote Eylandt in 2006) were soon eliminated [1]. We present a case of dengue acquired in a region assumed to be free of dengue vectors.

On 20 July 2010, a 34-year-old man presented to the public hospital in Darwin on the north coast of the NT with a one day history of rash and fever, retro-orbital headache, and prominent myalgia. He denied any travel outside the Northern Territory in the previous month. On examination he was febrile (38.3°C) and had an extensive blanching erythematous rash on his trunk, back, and upper and lower limbs.

A serum sample referred to PathWest Laboratory Medicine WA and tested for NS1 antigen by a commercial enzyme immunoassay (Bio-Rad Platelia Dengue NS1 Ag) was strongly positive. Dengue virus (DENV) was detected using a type-specific semi-nested polymerase chain reaction (PCR). The first round used consensus outer primers that detect all four dengue serotypes, followed by a second round using type-specific inner primers [6]. A later sample showed a rise in haemagglutination inhibition antibody titre to >1∶640, and was positive for IgM to DENV.

Samples were also sent to a second reference laboratory (Queensland Health Forensic and Scientific Services) for confirmation and sequencing of the envelope gene. Nucleotide sequencing and phylogenetic analysis of the DENV-1 E gene (1,485 base pairs) was carried out comparing the NT 2010 sequence with 53 other sequences derived from local, imported, or global origins (A. Pyke, unpublished data). The NT 2010 sequence (GenBank accession number HQ871946) was designated within DENV-1 genotype IV, which contains predominantly Pacific strains, but initial nucleotide comparisons did not reveal highly similar sequences amongst other available strains. However, in September 2010, analysis of virus obtained from a symptomatic traveller recently returned from Bali, Indonesia (Bali 2010a, Figure 1), to Cairns revealed 100% homology with the NT 2010 sequence, strongly suggesting that Bali may have been a likely geographical source for the NT 2010 strain. Of note, several other strains originating from Bali had been imported into Queensland during 2010 (Bali 2010b to Bali 2010f, Figure 1) that were distinctly different from each other and were implicated in both genotype IV and genotype I.

**Figure 1:**
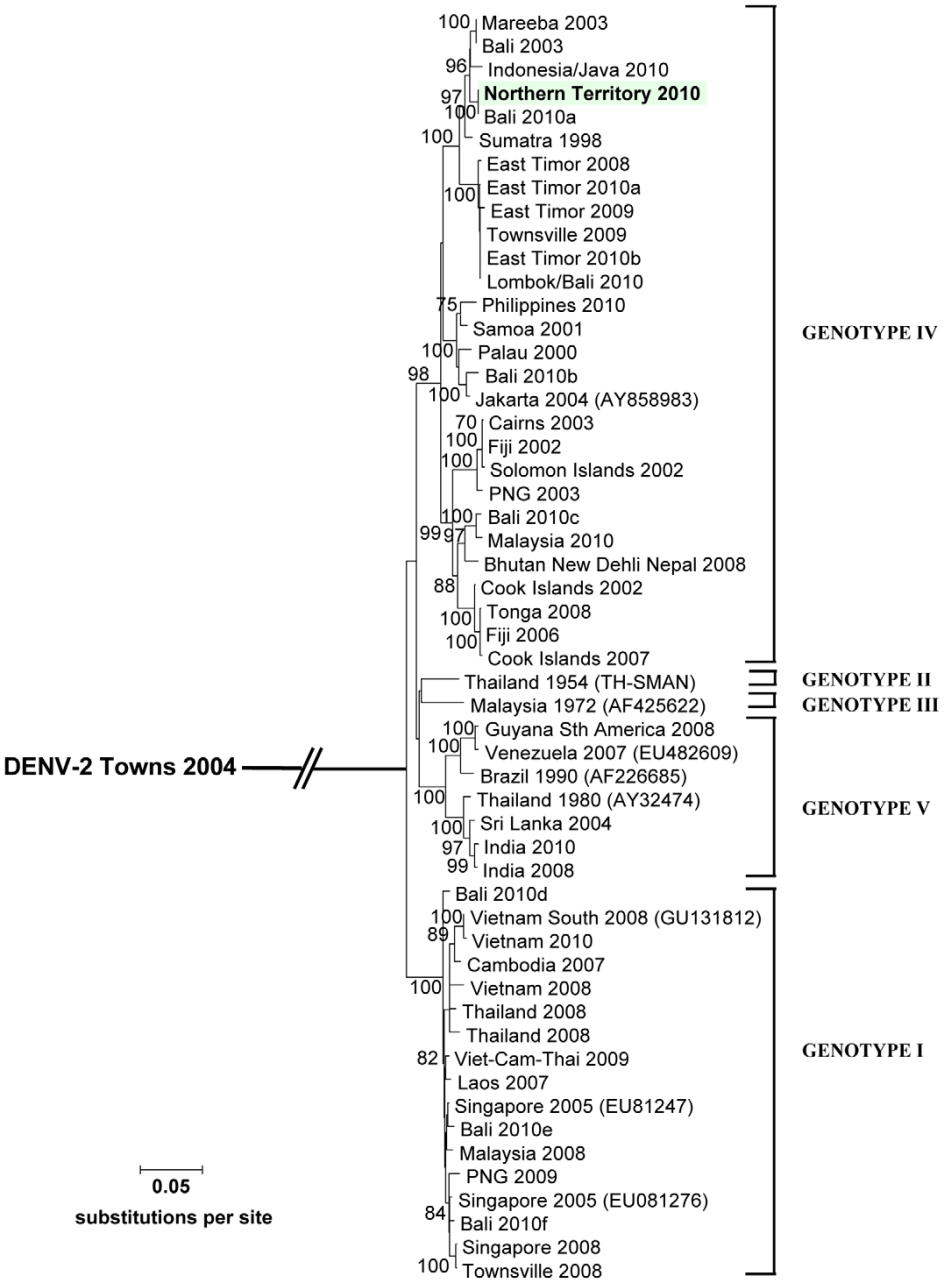
Maximum likelihood tree comparing DENV-1 complete E gene sequences (1485 base pairs). Strain names and respective genotypes are given including locally transmitted viruses (Townsville, Cairns, and Mareeba). GenBank accession numbers of retrieved global strains are shown in brackets. Bootstrap support values derived from 1,000 replicate NJ trees are represented for principal nodes >70%. DENV-2 strain Townsville 2004 was used as an outgroup control.

**Figure 2:**
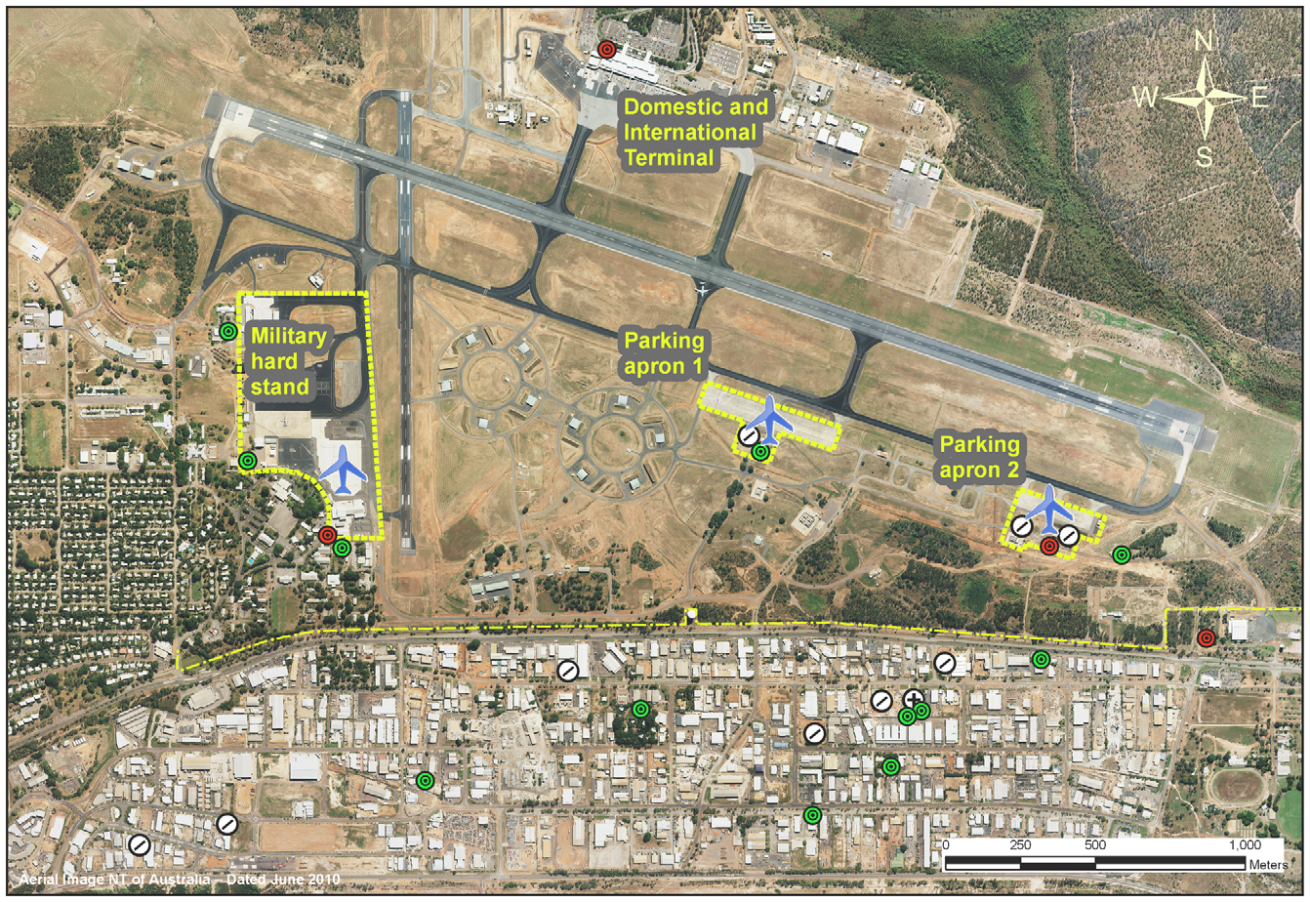
Local area of the case's workplace. Shown are: the case's workplace (cross in circle), containerised cargo delivery locations (dash in circle), apron boundaries (dashed line), southern airport boundary (dot and dash line), airplane arrival areas (airplane symbol), routine adult mosquito traps (red circle), and extra case adult mosquito traps (green circle). Image supplied courtesy of Northern Territory Government.

The case gave consent for the details of his illness to be published. He lived in an outer suburb of Darwin and worked in an industrial zone on the southern boundary of the Darwin Airport, which functions as both a civilian and military airport (Figure 2). Local shipping companies and major local removal companies were asked to provide the details of deliveries from overseas or north Queensland (where there are dengue vectors) to within 500 metres of the case's workplace or home suburb in the three weeks prior to his illness. These were inspected for mosquito breeding sites.

Eight mosquito larval surveys, four egg trapping surveys using ovitraps, and 12 adult mosquito trapping surveys using Biogents and CO2-baited traps were carried out at the case's residence, places visited, workplace, and nearby premises, including four locations within the airport's southern boundary from 5 August until the early wet season rains had been established (October). These failed to reveal any dengue vectors. All recent adult mosquito collections in Darwin, part of the routine mosquito surveillance program, were re-examined for any evidence of exotic mosquitoes but none were found. Public Health Units in Queensland were contacted to establish the nature of any recent outbreaks of type 1 dengue.

During July, Darwin Airport received approximately 20 flights from north Queensland and 50 international flights per week including 14 per week from Bali [7]. These flights park on the northern side of the airport, some 2.5 km from the case's workplace and separated by the open expanse of runways. A further 14 military aircraft flights arrived at the Royal Australian Air Force (RAAF) base from several overseas destinations between 1 and 15 July, including two flights of C-130 aircraft from Bali (9 July and 13 July), the second of which coincided with the likely date of acquisition of the case. These flights unloaded on the military hardstand 1,800 m from the case's workplace but may have subsequently opened doors at the parking apron 700 m away (Figure 2).

Despite heightened awareness and active case-seeking, no further cases were detected in Darwin.

## Discussion of Case

Prior to this case, there had not been a documented case of locally acquired dengue in the NT since the 1950s. We consider it highly unlikely that there were undetected *Ae. aegypti* mosquitoes at any of the places the patient had worked, resided, or visited, given the degree of trapping that was undertaken and the sensitivity of this method for finding *Ae. aegypti* in the past [8]. Therefore it is presumed that the infection was acquired from an infected mosquito imported from overseas or northern Queensland.

The proximity of the case's workplace to the RAAF base, and the arrival there of a C-130 aircraft from Bali just prior to the most likely date of acquisition, raise the possibility that it was acquired from an infective mosquito which escaped this aircraft.

Alternatively, it is possible that an *Ae. aegypti* mosquito infected in northern Queensland or overseas escaped from a commercial aircraft at the commercial terminal. However, the case worked about 2.5 km distant from the northern side of the airport and the intervening area is a ridge of open expanse including runways and taxiways (Figure 2), and the ecology of this mosquito species suggests it would be unlikely to cross such an open area.

Mosquito ecology would also suggest that air transport is associated with a higher risk of introducing a live infected mosquito than sea or road transport, and therefore the most likely source of dengue infection for this case is an infected mosquito arriving via an aircraft. The proximity of the case-patient's workplace to the RAAF base implicates that as a possible route, but the evidence is nevertheless circumstantial.

The Australian Quarantine and Inspection Service (AQIS) disinsection procedures are mandatory for all international aircraft arriving in Australia [9]. Disinsection should occur either at pre-embarkation or at the top of descent with an amount and type of insecticide spray specified for all aircraft types [9]. This is verified by an AQIS officer on arrival [9], as was the case here. It is possible that a mosquito harbouring inside covered cargo during the knockdown procedure survived the disinsection and escaped after arrival, either when the cargo was unloaded or after having been transported in the cargo to another location. It is also possible that the mosquito was harbouring in the wheel wells, which were not disinsected. Disinsection is not required in aircraft arriving from Queensland.

The genotyping of this virus was important in assisting in the investigation of the case. As the likely origin was only determined when a Bali-acquired case presented in another Australian state 6 weeks after this case, it demonstrated the value of centralising sequence data for viruses of public health significance. There is nevertheless always some uncertainty about the exact origin of viral strains. There were multiple strains emerging from Bali during the 2010 season, which might reflect the increase in travel to this destination, but also might mean that some of these strains were acquired from other locations but labelled as Bali strains, as Bali is often the last stopover before leaving South East Asia.

In summary, this is the first recognised case of locally transmitted dengue in the NT since the 1950s. We consider the most likely source of dengue to be a mosquito which alighted from a C-130 military aircraft arriving from Bali in early July and that this may be a case of “airport dengue” equivalent to previous reported cases of airport malaria [10], [11]. The case reinforces the need to continue strict disinsection of overseas aircraft and in monitoring mosquito populations around ports of entry. Health authorities and clinicians should be aware of this possible mode of introduction and transmission of dengue.

**Learning Points**

- Dengue can be transmitted to non-endemic regions through the transport of infected mosquito vectors in the same way as airport malaria.
- Clinicians should be aware that, with the increase in global travel, international trade, and military movements, dengue can appear in places far removed from regions where vectors are established.
- The centralisation of sequence data on viruses allows for comparisons between isolates and greatly assists in the analysis of transmission dynamics in viral diseases.
